# What Kills

**Published:** 1880-10

**Authors:** 


### WHAT KILLS.

In the school, as in the world, for more rust out than wear out. Study is most tedious and werrisome to those who study least. Drones always have the hardest time Grumblers make poor scholars, and their lessons are uniformly "hard and too long." The time and thought expended in shirking would be ample to master their tasks. Sloth, gormandizing and worry kill their thousands where over-study harms one. A curse rests on laziness and gluttony. By the very constitution of our being they are fitted to beget that torpor and despondency which chill the blood, deaden the nerves, enfeeble the muscles and derange the whole vital machinery. Fretting, fidgeting, *ennui* and anxiety are among the most common causes of disease. On the other hand, high aspiration and enthusiasm help digestion and respiration, and send an increased supply of vital energy to all parts of the body. Courage and work invigorate the whole system, and lift one into a purer atmosphere, above the reach of contagion. The lazy groan most over their "arduous duties," while earnest workers talk little about the exhausting labors of their profession. Of all creatures the sloth would seem to be the most worried and worn.—*The Sanatarian.*

